# On the Anodyne Virtue of Camphor

**Published:** 1801-08

**Authors:** John Ring

**Affiliations:** New Street, Hanover Square


### *On the anodyne Virtue of Camphor*; by Mr. Ring, *Member of the Royal College of Surgeons in London.*

To the Editors of the Medical and Physical Journal.

Gentlemen,

Having read in your Journal an account of the peculiar efficacy of camphor in a large dofe, as an anodyne in cafes of painful menftruation, I tried it, and found it deferving of every encomium it had received.

Previous to the exhibition of this medicine, the patient, who is a fervant, fuffered at every return of the catamenia the moft excruciating tortures; infomuch, that fhe was not only obliged to go to bed, but fhe ufed to beat herfelf, and was quite frantic, from the dreadful agony ſhe endured, for above four hours. —I directed ten grains of camphor, rubbed with an equal quantity of ſugar, to be taken in water on the acceſſion of the pain; and it acted like a charm, for in a quarter of an hour the pain was almoſt entirely removed.

She now takes it regularly at every monthly period, and with ſimilar advantage; ſo that ſhe is able, at thoſe times, to follow her uſual occupation without inconvenience.

*New Street, Hanover Square,* I am, &c.

